# Medically Serious and Non-Serious Suicide Attempts in Persons Aged 70 and Above

**DOI:** 10.3390/geriatrics1030023

**Published:** 2016-09-15

**Authors:** Stefan Wiktorsson, Petter Olsson, Margda Waern

**Affiliations:** Section for Psychiatry and Neurochemistry, Gothenburg University, 41345 Gothenburg, Sweden; petter.olsson@live.se (P.O.); margda.waern@neuro.gu.se (M.W.)

**Keywords:** suicide attempt, medically serious, older adults, depression, anxiety, antidepressant treatment

## Abstract

High rates of suicide are observed among older adults in many countries worldwide. In clinical settings, those who make a medically serious suicide attempt are generally considered to be at higher risk of subsequent suicide than those who make less serious attempts. Medically serious attempts in older clinical cohorts are, however, relatively understudied. The aim was to compare older adult suicide attempters (70+) who did or did not make medically serious attempts. We hypothesized, in line with the Interpersonal Model of suicide, that social problems and feelings of being a burden would be associated with medical seriousness. Participants (*n* = 101) were recruited in hospitals in the aftermath of a suicide attempt; they took part in an interview with a research psychologist. Attempters with (*n* = 28) and without (*n* = 73) medically serious attempts were compared. Major depression was common in both groups, and scores on the Geriatric Depression Scale did not differ. However, older adults who made medically serious attempts scored higher on the Brief Scale of Anxiety and lower on the Mini Mental State Examination than their peers who made less serious attempts. Medically serious attempters more often attributed the attempt to social problems as well as problems with functioning and autonomy, but perceived burdensomeness was not associated with seriousness. Findings may help to inform clinicians who meet and treat older suicidal persons.

## 1. Introduction

High rates of suicide are observed among older adults in many countries worldwide. A recent population-based study set in Denmark showed that an age over 55 years was the strongest predictor of subsequent suicide among emergency department patients who presented with a first suicide attempt [1]. In clinical assessments of suicide attempters, the use of a medically serious method is considered a particular marker of elevated risk for subsequent suicide. Older persons (50+) with medically serious attempts share many risk factors with those who die by suicide [2], further highlighting the need for qualified treatment and follow-up of older persons with medically serious attempts.

Hospital settings, which may be involved in the care of older adults after a medically serious attempt, include emergency and intensive care units, medical and surgery wards, geriatric wards, and psychogeriatric wards. Psychiatric services, primary care, and community services for older adults are often indicated after discharge. For the prevention of suicide in a high-risk patient group, we need to know more about older adults who make serious suicide attempts. While there are numerous studies comparing medically serious suicide attempters with their peers who make less serious attempts in younger cohorts [3,4,5,6], and one that includes both middle-aged and older attempters [7], studies with a specific focus on older adults are lacking. This is an area in need of study since factors associated with medically serious attempts in later life may differ from those demonstrated for younger patient cohorts considering the strong role of depression, physical illness, and functional disability [8] in the development of suicidal behavior in older adults. The aim of the current study was thus to compare clinical and psychosocial characteristics in older adult attempters (70+) with and without medically serious suicide attempts. Further, reasons for attempting suicide were compared in the two groups. Taking as a starting point the Interpersonal Theory of Suicide in Older People [9], we hypothesized that persons in the medically serious group would be more likely to attribute the attempt to social disconnectedness and feelings of burdensomeness than their peers who made less serious attempts.

## 2. Methods

### 2.1. Participants

**Figure 1:**
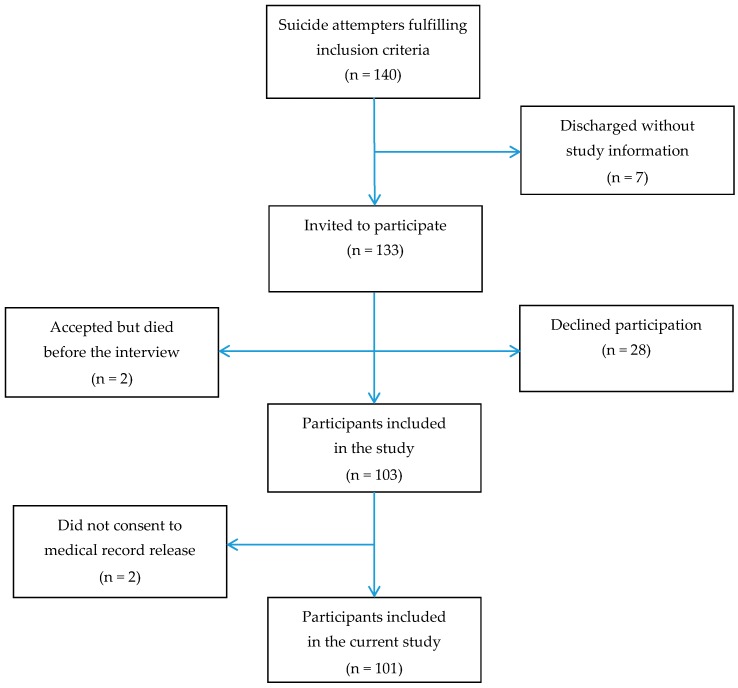
Participant flow chart of hospitalized attempters, aged 70 years and above.

Persons aged 70+ who were admitted to emergency wards in connection with a suicide attempt were asked to participate in the study [10]. A suicide attempt was defined as “a situation in which a person has performed actual or seemingly life-threatening behavior with the intent of jeopardizing his life, or to give the appearance of such an intent, but which has not resulted in death” [11]. Participants were recruited from three hospitals in the western part of Sweden during a three-year period (2003–2006). One hundred and three persons agreed to take part in the study (participation rate 77.4%). Two participants declined consent to a medical record release; the number of participants in the current study was thus 101 (47 men and 54 women). Mean age did not differ between women and men (80.5 vs. 78.7, t = −1.67, df = 99, p = 0.097). Participant flow is shown in detail in Figure 1.

### 2.2. Procedures

All interviews were carried out by the same psychologist (S.W.). The median time between the interview and the suicide attempt was eleven days [10]. Most interviews took place in the hospital wards, but 14 were performed after discharge. Twelve of these took place in the participant’s home, one at an outpatient department, and one in a nursing home.

Instruments included in the interview were:

**Geriatric Depression Scale (GDS).** To evaluate the burden of depression, the Swedish version of GDS with 20 yes/no questions was used [12]. This scale [13] is a screening instrument constructed to fit older adults proven to be a valid measurement for depression in this age group. In addition to the overall GDS score, we also analyzed responses to a single GDS item, the hopelessness item (“Do you think your situation is hopeless?”) due to its central relevance in the current study.

**Brief Scale for Anxiety (BSA).** The BSA [14] was used to measure the burden of anxiety. This scale is derived from the Comprehensive Psychopathological Rating Scale (CPRS) [15] and includes 10 items: inner tension, hostile feelings, hypochondriasis, worrying over trifles, phobias, reduced sleep, autonomic disturbances (both reported symptoms and observed signs), aches and pains, and muscular tension. There is a six-grade scale of response alternatives for each item. A modified version of the BSA was employed in the current study. The phobia item was excluded, yielding a maximum score of 54.

**Mini Mental State Examination.** To test cognitive functioning, the Swedish version of the Mini Mental State Examination (MMSE) [16] was administered.

**The Cumulative Illness Rating Scale for Geriatrics (CIRS-G).** Physical illness and disability was rated with the CIRS-G [17]. A person with a rating of 3 (severe/constant disability or uncontrollable chronic problems) or 4 (extremely severe illness or severe disability) in any of the 13 somatic organ categories was considered to have a serious physical illness/disability [18,19].

**Sense of Coherence (SOC).** The Swedish 29-item version of SOC [20] was used to examine to what extent participants found their lives meaningful, manageable, and comprehensible [21]. The SOC has a seven grade scale of response alternatives for each question, yielding a maximum score of 203. A high score means strong SOC. 

**Loneliness.** One single question (“Do you feel lonely?”) with a yes/no response was used to investigate perceived loneliness.

**Self-Reported Reasons for Attempting Suicide.** A single question, “Why did you attempt suicide?” was used to explore to what these older persons attributed their suicide attempt [9]. There were no prompts or follow-up questions, due to the frailty of the participants. The interviewer recorded the participants’ spontaneous responses by hand. Responses were analyzed and coded into themes by two independent researchers. After a consensus discussion, nine final themes were confirmed: somatic problems and pain, functioning and autonomy, psychological problems, social problems, lack of meaning, perceived burden, escape, wanting to die or sleep without a specific reason, and no memory or understanding of the suicide attempt. Participants could report more than one reason.

### 2.3. Diagnostics and Classification of Attempts

**Major and Minor Depression.** Major depression was diagnosed according to a symptom algorithm based on the Diagnostic and Statistical Manual of Mental Disorders 4th edition, DSM-IV [22]. Similarly, a symptom algorithm was used to determine participants with minor depression, in accordance with DSM-IV research criteria.

**Alcohol Use Disorder.** A lifetime prevalence of Alcohol Use Disorder (AUD) was determined if misuse or dependence was established from any of the following sources: interview data, case records, and the regional hospital discharge register [23]. A broad definition of AUD was used to include all those with previous or current problematic alcohol use.

**Dementia.** Dementia was diagnosed according to DSM-III-R [24]. A dementia diagnosis requires (A) impairment in short- and long-term memory and (B) at least one of (B1) impairment in abstract thinking, (B2) aphasia, (B3) apraxia, (B4) agnosia, or (B5) personality change. Procedures have been previously described in detail [25].

### 2.4. Classification of Suicide Attempts

Methods were denoted as non-violent (overdose, poisoning) or violent (hanging, cutting, drowning, and other violent methods) [26]. Further, for the purpose of this study, we defined medically serious suicide attempts (MSSA) with a definition similar to that applied in the Canterbury studies in New Zealand and recent Israeli studies [2,27]. A suicide attempt was classified as medically serious if it warranted hospitalization for at least 24 h and treatment in a specialized unit (including intensive care unit, hyperbaric unit, and burn unit), surgery under general anesthesia (e.g., tendon rupture), or extensive medical treatment (beyond gastric lavage, activated charcoal, or routine neurological observations), including antidotes for drug overdose. We could not apply the telemetry criterion used in previous studies as we did not have access to these data. Persons who were hospitalized for more than 24 h after a suicide attempt by hanging were also considered to have an MSSA, due to the high risk of fatality associated with this method [2]. A medically non-serious suicide attempt (NMSSA) was defined as a suicide attempt that did not meet any of the above criteria.

### 2.5. Statistics

The *t*-test was used to calculate differences in means. Fisher’s exact test was applied to test for differences in proportions. Conditional logistic regression models were constructed including all significant and near-significant variables from the pairwise analyses. All statistical tests were two-sided. Statistical significance was determined when *p*-values were less than 0.05. Analyses were carried out using IBM SPSS, Statistics for Windows, Version 22.0. (IBM Corp, Armonk, NY, USA).

### 2.6. Ethics

Written consent was acquired from all participants following oral and written information about the study. Participants were assured that they had the right to withdraw from the study at any time. The study was approved by the Research Ethics Committee at the University of Gothenburg, S 063-03.

## 3. Results

### 3.1. Suicide Methods and Medical Severity

Most (78%) of the participants used non-violent methods. Violent methods were employed by 26%. Four persons combined both violent and non-violent methods. Benzodiazepines were ingested in almost a third of all of the cases. The widely prescribed hypnotics zopiclone and zolpidem were used in around 15% of the attempts each. Twenty-eight of the attempts (17 by women and 11 by men) were classified as medically serious and 73 as non-medically serious (37 by women and 36 by men). Overdoses of one or more medications were involved in over two-thirds of the MSSAs (19 out of 28). Half of the cuttings (6 out of 11) were classified as MSSA. One of the five hangings was not considered medically serious because there were no visible signs (neither abrasions, contusions, or edema of the neck nor subconjunctival or skin petechiae) and the participant’s description of the act indicated low lethality. None of the attempts involved gunshot wounds. Details are shown in Table 1.

### 3.2. Psychosocial and Clinical Characteristics in Persons with MSSA vs. NMSSA

No gender difference was observed between those with and those without an MSSA (women 60.7% vs. 50.7%, *p* = 0.366), nor did mean age between the two groups (79.5 vs. 79.8, *t* = 0.22, df = 99, *p* = 0.824). No differences were observed regarding psychosocial characteristics (Table 2). Table 2 shows further that proportions with major depression did not differ between the two groups. The same applied to other diagnoses including minor depression, history of alcohol use disorder, and dementia. Three-quarters of those with an MSSA and half of those with an NMSSA had an ongoing prescription for one or more antidepressants at the time of the suicide attempt. The difference did not reach significance (*p* = 0.070). Geriatric Depression Scale scores were very similar in persons with and without an MSSA. However, higher anxiety ratings were observed in those with an MSSA. The two groups also differed regarding cognitive capacity as measured by the MMSE; lower scores were found among those with an MSSA.

### 3.3. Reasons for Attempting Suicide in Persons with MSSA vs. NMSSA

Older adults with MSSA were more likely to attribute the attempt to social problems compared to their peers with NMSSA, but this was not the case for being a burden to others (Table 3). Attributing the attempt to problems involving functioning and autonomy was associated with seriousness status. Non-serious attempters were more likely to report that they wanted to die or sleep, without giving a specific reason for the suicide attempt. Such a response was recorded in none of those with an MSSA.

### 3.4. Regression Models

In a conditional logistic regression including all significant and near-significant variables from the pairwise analyses, the MMSE score remained a significant predictor of seriousness status (OR = 0.81, 95% CI: 0.71−0.93, Wald = 9.01, df = 1, *p =* 0.003). Social relationship problems showed a near-significant association in this model (OR = 3.75, 95% CI: 0.99−14.06, Wald = 3.83, df = 1, *p =* 0.050). Adding sex and age to the model did not change results. The MMSE score remained a significant determinant of medical seriousness (OR = 0.83, 95% CI: 0.71−0.97, Wald = 5.57, df = 1, *p =* 0.018) in a model that also included the proxy brain damage variable.

## 4. Discussion

### 4.1. Findings

While major depression was common in both groups, and similar scores were observed on the Geriatric Depression Scale, older adults who made medically serious suicide attempts scored higher on the Brief Scale of Anxiety and lower on the MMSE than their peers who made less serious attempts. To our knowledge, this is the first study to investigate reasons for attempting suicide in older adults with medically serious attempts. In line with one of our expectations, those with an MSSA more often attributed their attempts to social problems. Contrary to our hypothesis, we did not find support for an association between perceived burdensomeness and medical seriousness.

The mean age of the participants in our study was 80 years, and previous studies focusing on attempters in this age group are lacking for direct comparison. Results regarding sociodemographic and clinical characteristics can be compared with the New Zealand-based study of [2], which focused on a somewhat younger cohort (attempters aged 55 and above, mean age 63 years). Rates of affective illness and lifetime substance use disorders were very similar in the two cohorts, but there were notable differences in terms of gender (over half of those with medically serious attempts in our study were women, compared to 8% in the New Zealand study), serious physical illness (75% in our study as compared with 27% in the New Zealand study), and previous history of attempted suicide (one-third in our study compared to almost three-quarters in the New Zealand study). These disparities may in part be related to the higher age of the participants in our study, but cultural differences may also be at play.

We found no difference in Geriatric Depression Scale scores between those with and without an MSSA. This parallels results from a previous study involving a younger sample (aged 16–71 years) in which similar scores on the Beck Depression Inventory were observed for both groups of suicide attempters [28]. In our study, older persons with an MSSA had higher levels of anxiety than their peers with an NMSSA. We found no study comparing levels of anxiety in persons with and without an MSSA for comparison, and the role of anxiety in serious suicidal behavior needs further elucidation. A relevant clinical question is whether symptoms of anxiety might be, in some cases, driven by delirium related to the attempt itself. This is less likely, however, considering the fact that interviews were scheduled first after patients were medically stabilized, with a mean of 11 days after the attempt.

The group with an MSSA in the current study had a mean MMSE score indicating possible mild cognitive impairment. Reduced cognitive performance might have impacted on problem solving skills, contributing to the seriousness of the suicidal process. It is not clear whether reduced cognitive function might be related to the attempt itself. This was suggested in our recent prospective study that used a somewhat different definition of medical seriousness [29]. In the current study, we used a proxy variable (at least 24 h in the Intensive Care Unit (ICU)) in order to take into consideration the possible effects of attempt-related brain damage. The relationship between a lower MMSE score and attempt seriousness remained in the regression model adjusted for the proxy variable. Regardless of whether compromised cognition is a result of the attempt, an ongoing depression, or neurovascular or degenerative disease, physicians and staff need to take keep reduced cognition in mind when caring for patients with medically serious suicide attempts. 

Relatively few of the participants in our study attributed the attempt to being a burden to others, and proportions were similar in those with an MSSA and an NMSSA. The latter finding contrasts with results from a recent study involving a broader age group (42 years and above) in which patients with low-lethality attempts were more likely to report feelings of being a burden to others compared to those with high lethality attempts [7]. The studies are not directly comparable to those who reported being a burden in our study, who were responding to an open question about why they attempted suicide. Patients may have responded differently had we asked specifically whether they had feelings that they were a burden to others. In the current study, we did find a relationship with functioning and autonomy. However, this did not hold up in the final regression model, suggesting that this attribution might not be an independent determinant of attempt seriousness in older adults.

Results may not apply to other geographical settings as methods employed in suicidal behavior vary widely throughout the world. Furthermore, being rescued is a prerequisite for survival in connection with a medically serious attempt. One attempter might survive a serious attempt, while another who makes an attempt with similar lethality in another setting may not have the same chance of getting fast and effective medical attention.

### 4.2. Methodological Considerations

Strengths of the study include the careful characterization of symptoms by a single interviewer and the fact that the attempters were given an opportunity to describe in their own words the reasons for the attempt. An important limitation is the size of the study. It is reasonable to assume that some of the non-significant associations shown here might prove significant in a study with larger power. For example, regarding the finding that 75% of those with medically serious attempts were on antidepressants compared to 53% in the non-serious group, a post hoc power calculation (set at 80% power) showed that 140 participants would be needed to demonstrate a significant (0.05) group difference. A considerably larger sample size (348) would be required to demonstrate a significant difference for the Sense of Coherence score. Another consideration is that our study is hospital-based, and older persons who attempt suicide but do not present at hospital are missed. On the other hand, with the exception of the attempted hangings, most of those with medically serious attempts would probably not survive had they not received hospital care. They would have died by suicide, and would thus not be recruited in a study of suicide attempters set outside the hospital.

It is important to remember that the primary focus of the classification system employed in this study was on the medical consequences of the attempt, as determined by the clinical management. Local routines and the availability of specialized services will impact on this. For example, the threshold for intensive care may differ among facilities. Further, the individual physician’s degree of experience and personal preferences will impact treatment decisions (for example, whether or not to employ an antidote). We tried to compensate for this by applying a conservative interpretation of seriousness. It is important to point out that, while medically serious attempts tend to be related to higher suicide risk, medical seriousness in and of itself cannot be construed as a measure of high risk. An attempt may turn out to be medically serious despite a relatively low grade of intention. This might be particularly relevant for the attempts of older persons, where physical frailty and polypharmacy may determine the medical seriousness of an attempt. Medical seriousness of a suicide attempt is a single dimension of suicidal behavior. An avenue for future research would be to use a comprehensive definition [6] that includes two other salient factors (the degree of objective intent and the potential fatality of the method employed). This type of approach could be combined with an in-depth study of reasons for suicide attempts in older adults. Research needs to be carried out in diverse geographical regions to inform culturally appropriate interventions for older people at high risk of suicide.

### 4.3. Implications

As the majority of older adults who make serious suicide attempts have comorbid serious physical illnesses, our study’s findings have implications not only for psychiatric services but also for those who care for older persons with somatic ill-health. Liaison psychiatry services can play an important role in securing appropriate care in the aftermath of a suicide attempt [30]. Asking suicidal persons about the reasons for their attempts can help to inform person-centered interventions [31]. The finding that three-quarters of those with medically serious attempts were already being treated with antidepressants is a reminder that the prevention of serious suicidal behavior in older adults may require enhanced treatment including both pharmacological and psychosocial modalities.

## 5. Conclusions

Older suicide attempters with an MSSA had higher anxiety ratings and lower cognitive function scores than their peers who made less serious attempts. Further, they were more likely to attribute the attempt to social problems as well as problems with functioning and autonomy. Results from this study may help to inform clinicians, as greater knowledge about the factors associated with serious attempts could provide a basis for a more person-centered treatment.

## Figures and Tables

**Table 1:** Suicide attempt methods among older adult suicide attempters (*n =* 101) with and without medically serious attempts (MSSA).

Suicide Methods	Medically Serious Attempt		Total
	No (*n =* 73); *n* (%)	Yes (*n =* 28); *n* (%)	(*n =* 101); *n* (%)
**Non-Violent Methods (Overdose/Poisoning)**			
Sedatives and hypnotics			
Bensodiazepines	23 (31.5)	9 (32.1)	32 (31.6)
Zopiclone	12 (16.4)	4 (14.2)	16 (15.8)
Zolpidem	13 (17.8)	2 (7.1)	15 (14.8)
Others	6 (8.2)	3 (10.7)	9 (8.9)
Antidepressants	7 (9.6)	1 (3.6)	8 (7.9)
Antipsychotics	1 (1.4)	2 (7.1)	3 (2.9)
Lithium	0 (-)	1 (3.6)	1 (0.9)
Analgesics	1(1.4)	2 (7.1)	3 (2.9)
Opioids	4 (5.5)	4 (14.3)	8 (7.9)
Paracetamol	3 (4.1)	2 (7.1)	5 (4.9)
Other substances	14 (19.2)	3 (10.7)	17 (16.8)
**Violent methods**			
Drowning	2 (2.7)	0 (-)	2 (1.9)
Hanging	1 (1.4)	4 (14.3)	5 (5.0)
Strangling	7 (9.6)	0 (-)	7 (6.9)
Suffocation	1 (1.4)	0 (-)	1 (0.9)
Cutting	5 (6.8)	6 (21.4)	11 (10.9)

**Table 2:** Characteristics of older adult suicide attempters (*n =* 101) with and without an MSSA.

Characteristics	Medically Serious Attempts		Test Result ^1^ *p*-Value
	No (*n =* 73); *n* (%)	Yes (*n =* 28); *n* (%)	
**Psychosocial Characteristics**			
Living alone	52 (71.2)	16 (57.1)	0.236
Married/living with a partner	21 (28.8)	12 (42.9)	0.236
Education, mandatory only	40 (54.8)	17 (60.7)	0.658
Loneliness	41 (56.2)	17 (60.7)	0.823
Family history of suicide	6 (8.2)	4 (14.3)	0.458
**Clinical Characteristics**			
Major depression	46 (63.0)	21 (75.0)	0.348
Minor depression	19 (26.0)	7 (12.5)	1.000
Alcohol use disorder	22 (30.1)	5 (17.9)	0.315
Dementia	4 (5.5)	4 (14.3)	0.212
Serious physical illness	42 (57.5)	21 (75.0)	0.116
History of psychiatric treatment	41 (56.2)	18 (64.3)	0.506
Previous suicide attempt(s)	26 (35.6)	10 (35.7)	1.000
Antidepressant prescription	39 (53.4)	21 (75)	0.070
Hopelessness ^2^	39 (56.5)	15 (55.6)	0.932
**Rating Scales**	Mean (SD ^3^)	Mean (SD)	Test Result ^4^
Geriatric Depression Scale, (*n =* 96)	9.5 (2.7)	9.9 (2.8)	*t =* −0.76, df = 94, *p =* 0.447
Brief Scale of Anxiety, (*n =* 97)	8.6 (5.3)	11.4 (6.3)	*t =* −2.27, df = 95, *p =* 0.025
Sense of Coherence, (*n =* 87)	131.3 (20.8)	123.9 (26.9)	*t =* 1.31, df = 85, *p =* 0.193
Cumulative illness rating scale, (*n =* 101)	9.6 (4.4)	9.1 (3.2)	*t =* 0.63, df = 99, *p =* 0.532
MMSE ^5^, (*n =* 96)	26.0 (3.5)	23.7 (4.3)	*t =* 2.70, df = 94, *p =* **0.008**

^1^ Fisher’s exact test; ^2^ Missing data for five participants; ^3^ SD stands for standard deviation; ^4^ *t*-test; ^5^ Mini Mental State Examination.

**Table 3:** Self-reported reasons for attempting suicide by seriousness status.

Reasons	Medically Serious Attempts		Test Result ^2^ *p*-Value
	No ^1^ (*n =* 72); *n* (%)	Yes (*n =* 28); *n* (%)	
Social problems	6 (8.3)	7 (25.0)	**0.043**
Being a burden to others	9 (12.5)	4 (14.3)	0.753
Functioning and autonomy	13 (18.1)	11 (39.3)	**0.037**
Somatic problems and pain	12 (16.7)	4 (14.3)	1.000
Psychological problems	16 (22.2)	8 (28.6)	0.603
Lack of meaning	6 (8.3)	2 (7.1)	1.000
Escape	19 (26.4)	10 (35.7)	0.462
Wanted to die or sleep ^3^	13 (18.1)	0 (0)	**0.017**
No memory or understanding	11 (15.3)	3 (10.7)	0.752

^1^ Missing data for 1 participant; ^2^ Fisher’s exact test; ^3^ Without any specific reason.
